# MACC1 Hyperactivates Receptor Tyrosine Kinase Signaling Through Phosphorylation-Dependent Adaptor Activity in Colorectal Cancer Cells

**DOI:** 10.3390/biom16091267

**Published:** 2026-09-02

**Authors:** Fabian Zincke, Dennis Kobelt, Susan Kläger, Fiona Pachl, Gerrit Erdmann, Mathias Dahlmann, Wolfgang Walther, Bernhard Küster, Ulrike Stein

**Affiliations:** 1 Experimental and Clinical Research Center, Charité—Universitätsmedizin Berlin, and Max-Delbrück-Center for Molecular Medicine, Robert-Rössle-Straße 10, 13125 Berlin, Germany; fabianzincke@web.de (F.Z.); dennis.kobelt@epo-berlin.com (D.K.); mathias.dahlmann@epo-berlin.com (M.D.); wowalt@mdc-berlin.de (W.W.); 2 German Cancer Consortium (DKTK), Neuenheimer Feld 280, 69120 Heidelberg, Germany; klaeger.susan@gene.com (S.K.); kuster@tum.de (B.K.); 3 Chair of Proteomics and Bioanalytics, Technical University Munich, Emil-Erlenmeyer-Forum 5, 85354 Freising, Germany; fiona.pachl@astrazeneca.com; 4 NMI TT GmbH, Markwiesenstraße 55, 72770 Reutlingen, Germany; gerrit.erdmann@gmx.net

**Keywords:** MACC1, colorectal cancer, metastasis, receptor tyrosine kinase signaling, adaptor protein, tyrosine phosphorylation, protein–protein interaction, signal transduction

## Abstract

Understanding the mechanisms of metastasis is one of the most pressing issues in cancer therapy. Metastasis-associated in colon cancer 1 (MACC1) is an important biomarker and functional driver of tumor progression and metastasis. However, the molecular mechanisms underlying its activity remain incompletely understood. Here, we demonstrate that MACC1 acts as an important adaptor protein that promotes hyperactivation of receptor tyrosine kinase (RTK) signaling pathways in colorectal cancer (CRC) cells. Based on mass spectrometry-based interactomics, we identified key MACC1 interactors, including GRB2, SHP2, SHC1, and STAT5B, that preferentially associate with tyrosine-phosphorylated residues Y365, Y379, and Y789. Site-directed mutagenesis of Y379 and Y789 reduced MACC1-induced migration, proliferation, and ERK phosphorylation. Using digital Western blotting (DigiWest), we observed a broad MACC1-dependent hyperactivation of downstream signaling effectors, including MEK, ERK, β-catenin, SRC, FAK, CREB, and VASP. Targeting MACC1-induced signaling with clinically relevant inhibitors effectively reversed MACC1-driven clonogenicity. Our findings support a role for MACC1 in promoting hyperactivation of RTK-associated signaling and reveal pharmacological vulnerabilities of potential relevance to metastasis-prone cancers characterized by elevated MACC1 expression.

## 1. Introduction

Cancer metastasis remains the leading cause of cancer-related deaths. In contrast to positive developments in curing primary cancers, metastases mostly demonstrate resistance to current therapeutic interventions [1]. Consequently, understanding the mechanisms behind metastasis is critical to developing more effective therapies. Hence, identifying biomarkers that not only determine patients at high risk but also drive metastasis and serve as therapeutic targets has become increasingly important in clinical oncology [2,3]. One such biomarker is MACC1 (metastasis-associated in colon cancer 1): a gene initially identified as upregulated in CRC tumors compared to normal colon tissue, and now known as a strong prognostic biomarker and a key driver in tumor progression and metastasis formation in several tumor entities [4].

MACC1 promotes multiple malignant processes, including migration, invasion, cell proliferation and colony formation, while enhancing tumor growth and metastasis in various animal models. Notably, MACC1 acts as a transcriptional activator of MET, driving downstream MEK/ERK and PI3K/AKT signaling. It further plays an influential role in epithelial-to-mesenchymal transition (EMT), conferring stem cell-like properties, chemoresistance, stemness, metabolic reprogramming, angiogenesis and lymphangiogenesis, apoptosis, immune response pathways, and cell cycle transition via different signaling pathways (e.g., PTEN/AKT, PI3K/AKT, MEK/ERK, and STAT) and expression of target genes [5,6].

MACC1’s structural features, including its SH3 domain, death domains (DDs), and various phosphorylation sites, support its role in protein interactions and signal transduction [4,7,8,9,10,11,12]. Given the critical role of MACC1 in multiple aspects of cancer biology, it holds significant potential for therapeutic intervention by targeted cancer therapies. The silencing of MACC1 or mutations in the SH3 domain, PxxP motif, and specific tyrosine phosphorylation sites on MACC1 impair its function in both in vitro and in vivo models, highlighting its therapeutic potential, particularly in metastatic cancer treatment [4,13].

In this study, we aimed to further elucidate the molecular mechanisms by which MACC1 regulates key signaling pathways involved in cancer progression and metastasis with implications for the design of new cancer therapies. Therefore, we investigated phosphotyrosine (pY)-dependent protein–protein interactions of MACC1 and their consequential influence on the MACC1 signaling landscape. We found that MACC1 interacts with key signaling molecules and confers a hyperactivated signaling phenotype to cancer cells which could be disturbed by the mutation of pY-interaction sites and by various inhibitors of known targets within MACC1-dependent signaling. This reveals new functions of MACC1 based on protein–protein interaction and identifies pharmacological vulnerabilities associated with MACC1-dependent signaling that may inform future therapeutic strategies for patients with metastatic disease or at high risk of metastasis.

## 2. Materials and Methods

### 2.1. Generation of Stably Transduced and Site-Directed Mutated Cancer Cells

Human cancer cell lines were either purchased from the American Type Culture Collection (ATCC, Manassas, VA, USA) or the German Collection of Microorganisms and Cell Culture (Leibnitz Institute DSMZ, Braunschweig, Germany) and cultivated in either DMEM or RPMI1640 medium (Thermo Fisher Scientific, Waltham, MA, USA) supplemented with 10% fetal bovine serum (FBS; Bio & Sell, Feucht, Germany) at 37 °C in a humidified incubator with 5% CO_2_.

1.5 × 10^7^ HEK cells were seeded and transfected after 24 h with a mixture of 2.85 mL serum-free medium, 90 μg polyethylenimine, 30 μg lentiviral helper plasmids (20 μg psPax2, 10 μg pMD2.G) and 20 µg coding lentiviral genomic plasmid, incubated for a total of 25 min at room temperature and added to the plates. After 48 h, the supernatant was collected, filtered (0.45 μm), and loaded onto a 20% sucrose cushion, followed by centrifugation at 28,000 rpm (max. 141,000× *g*, SW 28 rotor) for 4 h at 4 °C. The viral particles were resuspended in 500 μL sterile PBS and stored at −80 °C. Cells were transduced with an MOI less than 10, and after 24 h, the medium was replaced. GFP expressing cells were sorted by FACS.

To create MACC1-GFP overexpression and GFP control cells (HCT116/MACC1-GFP and HCT116/GFP), the plasmid RC224774L2 (Origene, Rockville, MD, USA) was used. Tyrosine mutant cell lines were generated by site-directed mutagenesis (SDM) using the QuikChange Lightning Multi Site-Directed Mutagenesis Kit (Agilent, Santa Clara, CA, USA) following the manufacturer’s instructions with the following primers: Y379F fwd—GGAATTTATGGACCCAAATTTATCCATCCCAGTTTTACTG and Y789F fwd—ATGATGTGGAAACCTGCCTTTGATTTTCTGTATACCTGGA.

Transfecting SW480 cells (an MACC1 low expressing cell line) with pcDNA3.1-V5-His and pcDNA3.1-MACC1-V5-His (Thermo Fisher Scientific) yielded SW480/ev control cells and SW480/MACC1 cells with stable MACC1 overexpression. Positive clones were selected using neomycin treatment. Stable overexpression was confirmed by monitoring MACC1 mRNA levels via quantitative real-time PCR (qRT-PCR).

### 2.2. Protein Extraction and Standard and Digital Western Blotting (WB)

Cells were harvested and lysed in ice-cold RIPA buffer (50 mM Tris, 150 mM NaCl, 1% NP-40, pH 7.5) with protease and phosphatase inhibitors (Roche Diagnostics, Risch, Switzerland). After a 30 min incubation and occasional vortexing, lysates were centrifuged at maximum speed for 45 min. Protein concentrations were determined using a Bicinchoninic Acid Protein Assay (Thermo Fisher Scientific). Denatured proteins were then separated by SDS-PAGE and analyzed using either standard WB or DigiWest.

For standard WB, 30 μg of protein was run on 10% Tris-HCl polyacrylamide gels in Tris/glycine/SDS buffer, transferred to PVDF membranes (Bio-Rad Laboratories Inc., Hercules, CA, USA) at 2.5 A and 25 V for 7 min employing the TransBlot^®^ TurboTM system (Bio-Rad), blocked with 5% BSA in Tris-buffered saline with Tween, and incubated overnight at 4 °C with primary antibodies (Supplementary Table S1). After applying HRP-conjugated secondary antibodies in Tris-buffered saline with Tween for 1 h at room temperature, protein bands were detected using WesternBright ECL (Advansta, San Jose, CA, USA) and CL-Xposure films (Thermo Fisher). The intensity of the bands was quantified using ImageJ (version 1.51j), and the signals were normalized to loading controls. Phosphorylated proteins were normalized to respective total protein levels. DigiWest was performed in collaboration with NMI TT Pharmaservices as described previously [14].

### 2.3. Co-Immunoprecipitation (Co-IP)

Cells (5 × 10^6^ per 10 cm dish) were incubated for 24 h and lysed in ice-cold IP-lysis buffer (20 mM Tris-HCl, pH 7.5, 150 mM NaCl, 0.1% NP40, 1 mM EDTA, 1% Triton X, with protease and phosphatase inhibitors). After a 30 min incubation on ice with intermittent vortexing, samples were centrifuged at 30,000× *g* for 45 min at 4 °C. Collected supernatants containing 2 mg of protein per sample were incubated overnight with 2 μg of the target antibody at 4 °C. Protein–antibody complexes were captured using 20 μL of protein G-agarose beads for 4 h at 4 °C on a shaker. The complexes were eluted with 30 μL LDS-buffer containing DTT (1:10) at 99 °C for 10 min. After final centrifugation, supernatants were analyzed by SDS-PAGE and WB.

### 2.4. Proliferation Assay

Cell proliferation was monitored using the IncuCyte^®^ ZOOM System (Essen BioScience, Ann Arbor, MI, USA). Cells (4 × 10^3^ per well) were seeded in 96-well plates and incubated for 24 h. Following treatment (0% FBS medium containing 20 ng/mL HGF), the plates were placed in the IncuCyte^®^ system for up to 144 h at 37 °C in a humidified incubator with 5% CO_2_. The system captured four images from each well every two hours, enabling real-time analysis of the cell-covered area. Data were processed and exported using IncuCyte^®^ ZOOM software (version 2016B).

### 2.5. Clonogenic Assay

The clonogenic assay was used to assess reproductive viability by evaluating the ability of individual cells to divide indefinitely, as determined by their capacity to form colonies [15,16,17]. HCT116/GFP and HCT116/MACC1-GFP cells were seeded in 24-well plates (200 cells/well) and allowed to adhere for 24 h. Following treatment, the plates were incubated in a humidified chamber at 37 °C with 5% CO_2_ for 7 days. After this incubation, the medium was replaced with a PBS solution containing 1% formaldehyde and 0.1% crystal violet to fix and stain the colonies. Colony area was quantified using the Colony Area plugin in ImageJ (version 1.51j).

### 2.6. Migration Assay

The Boyden chamber assay was utilized to assess the migratory capacity of cells. This assay measures the ability of cells to move in response to a chemotactic gradient through the pores of a trans-well membrane. Cells were serum-starved overnight. A total of 2.5 × 10^5^ cells (for 24-well plates) were plated in medium containing 2% FBS onto the trans-well insert, which was then placed into a well containing medium with 10% FBS. The plates were incubated for 24 h in a humidified chamber at 37 °C with 5% CO_2_. After incubation, cells from the bottom of the well and the bottom side of the membrane were trypsinized and collected by centrifugation at 5000 rpm for 5 min, and the number of viable cells was quantified using the CellTiter-Glo^®^ reagent (Promega, Madison, WI, USA). Luminescence was measured using the Infinite Pro multiplate reader (TECAN, Männedorf, Switzerland).

### 2.7. Assessment of Growth Factor Signaling

To investigate the impact of MACC1 on growth factor-mediated signaling, cells were seeded in 6-well plates at a density of 3 × 10^5^ cells per well and incubated for 24 h in a humidified chamber at 37 °C with 5% CO_2_. A serum starvation of 18 h was followed by growth factor treatment at 20 ng/mL (HGF, kindly provided by Prof. Dr. Walter Birchmeier, MDC Berlin, and EGF, Sigma-Aldrich, St. Louis, MO, USA) for 0, 1, 8, and 90 min. Removing the medium and washing the cells with ice-cold PBS stopped the treatment. Cells were then harvested in ice-cold RIPA buffer and processed for WB analysis as described previously.

### 2.8. Mass Spectrometry (MS) Screen

To identify interactors of tyrosine-phosphorylated MACC1, we performed a mass spectrometry (MS) screen. Bead-coupled peptides corresponding to all potential phosphotyrosine (pY) sites on MACC1 were used to capture binding partners from whole cell lysates. Peptides (Integrated DNA Technologies, Coralville, IA, USA; e.g., Y365—ATIWD(p)YIHKTT, Y379—IYGPK(p)YIHPSF, Y789—MWKPA(p)YDFLYT) were linked to NHS-Sepharose beads (Amersham Biosciences, Amersham, United Kingdom) as previously described [18]. SW480 and SW620 cells were lysed in IP buffer (0.8% NP40, 50 mM Tris-HCl pH 7.5, 5% glycerol, 1.5 mM MgCl_2_, 150 mM NaCl, 1 mM Na_3_VO_4_, 25 mM NaF, 1 mM DTT, phosphatase and protease inhibitors by Sigma-Aldrich). Lysates (5 mg/mL) were incubated with bead-coupled peptides for 30 min at 4 °C. After centrifugation and several washes with decreasing NP40 concentrations, proteins were eluted with LDS sample buffer (Invitrogen, Waltham, MA, USA) containing 50 mM DTT at 50 °C for 30 min. The eluates were alkylated with chloroacetamide (55 mM) and run via SDS-PAGE on a 4–12% NuPAGE gel (Invitrogen) for approximately 1 cm. Proteins were digested in-gel with trypsin.

The resulting peptides were analyzed using an Eksigent nanoLC-Ultra 1D+ system (Eksigent, Dublin, CA, USA) coupled to an LTQ-Orbitrap XL ETD (Thermo Fisher Scientific). Peptides were trapped on a self-packed C18 column (Reprosil-Pur C18-AQ 5 μm resin, Dr. Maisch GmbH, Ammerbuch, Germany) and separated on an analytical column (75 μm × 40 cm, self-packed with Reprosil-Pur C18-AQ, 3 μm resin; Dr. Maisch GmbH) with a 4–32% gradient in water plus 0.1% formic acid and 5% DMSO at a 300 nL/min flow rate. MS spectra were acquired at a resolution of 30,000 (*m*/*z* 400). Peptide identification and quantification were performed using MaxQuant software (version 1.5.3.30) by comparing MS/MS data to the Swissprot reference database [19,20]. Analysis parameters included trypsin as the protease, allowing two missed cleavages, with precursor and fragment ion tolerances of 10 and 20 ppm, respectively. The minimum peptide length was set to seven amino acids, with a false discovery rate of 1%.

### 2.9. Statistical Analysis

Data analysis and statistical calculations were performed using Microsoft Excel 2010 and GraphPad PRISM version 10.6.1. Groups of biological replicates were compared by one-way analysis of variance (ANOVA) with Tukey’s post hoc test (Figure 2B, Supplementary Figure S3) or two-way ANOVA with Tukey’s (Figure 2C) or Sidak’s post hoc test (Figure 5). All tests were two-sided with a 95% confidence interval (* = *p* < 0.05, ** = *p* < 0.01, *** = *p* < 0.001).

## 3. Results

### 3.1. MACC1 Interacts with Crucial Signaling Molecules via Specific pY-Sites

We have previously demonstrated that MACC1 is phosphorylated at specific tyrosine sites (Y673, Y695, Y793) by MEK1 and that inhibiting MEK1 or masking these sites restricts MACC1-induced functions, such as MET expression, cell motility, and proliferation in vitro as well as CRC metastasis formation in vivo [13]. To expand on this work and highlight the importance of MACC1 in malignant signaling processes, we investigated the phospho-interactome of MACC1.

**Figure 1:**
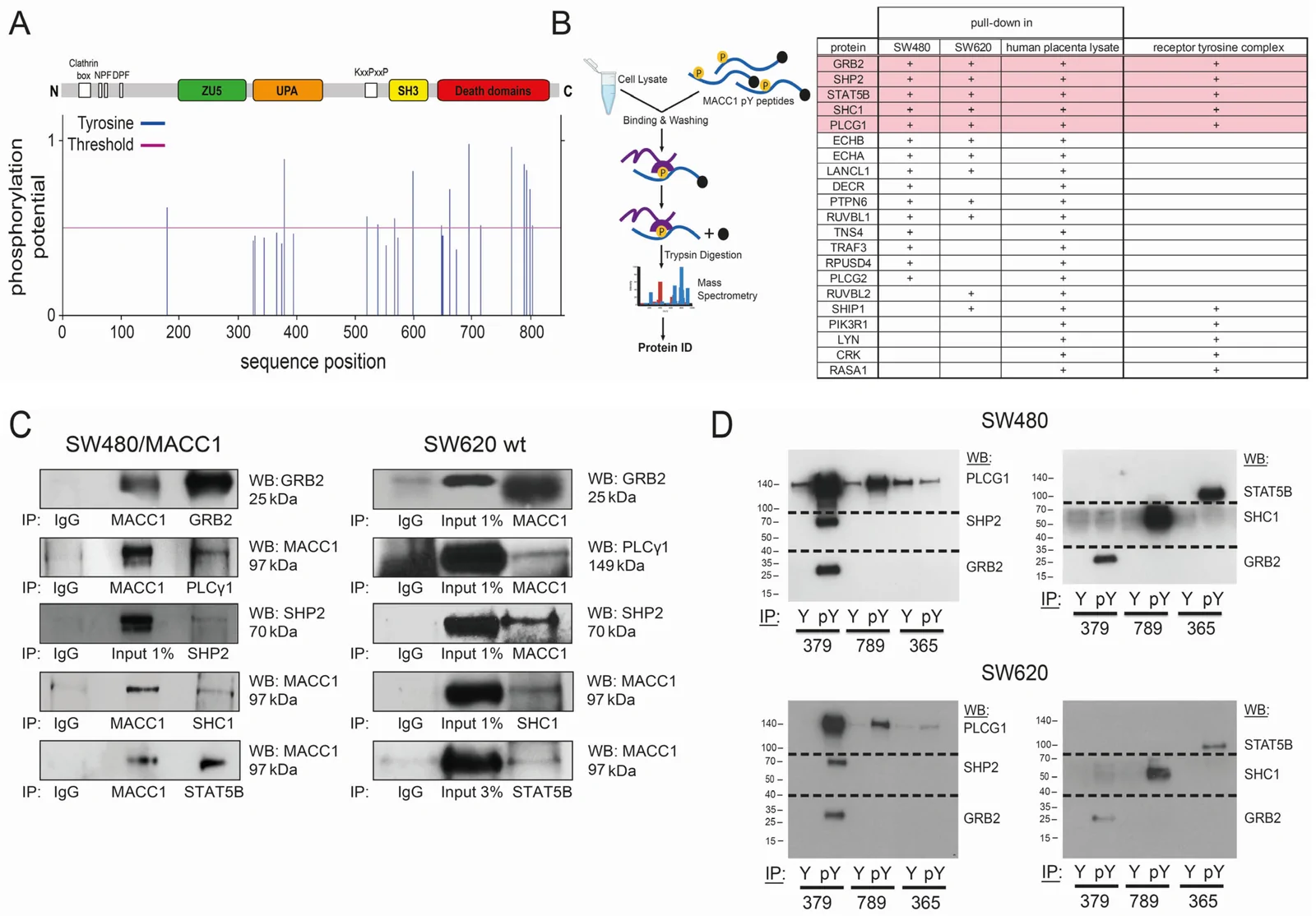
MACC1 interacts with crucial signaling molecules via specific pY-sites. (**A**) Domain architecture and phosphorylation potential (based on NetPhos 3.1 prediction [28] with MACC1 FASTA protein sequence) of MACC1 demonstrates several potential protein–protein interaction sites, including ZU5, UPA, SH3, and two DDs domains as well as (phospho-)tyrosine sites. (**B**) An MS screen identifies receptor tyrosine complex components as interactors of tyrosine-phosphorylated MACC1 in CRC (SW480) and CRC metastasis (SW620) cell lines, as well as in human placenta lysate. +—interactor identified in screen; Red—prioritized candidates (**C**) The most interesting interactions were validated by Co-IP in SW620 wild-type cells and SW480 cells ectopically overexpressing MACC1. Results show one representative example of at least two independent experiments. (**D**) Pull-down with phosphorylated or unphosphorylated peptides of the identified pY-interaction sites on MACC1 and subsequent WB shows preferential association of interactors with distinct phosphorylated MACC1-derived peptides.

To explore these interactions, pull-down assays were conducted using SW480 and SW620 CRC cell lines, along with human placenta lysate used as a broad-expression control, as 64% of all human proteins are reported to be expressed in placental tissue [21,22]. The assays involved bead-coupled peptides corresponding to all potential pY-sites of MACC1 (Figure 1B). Mass spectrometry (MS) analysis identified a broader set of candidate interactors associated with different MACC1 pY-containing peptides. For subsequent validation, we prioritized candidates that (i) were identified in CRC cell lysates, (ii) were consistently detected in both independent MS screens, and (iii) are established components of RTK complexes or associated signaling pathways. Based on these criteria, GRB2, SHP2, SHC1, STAT5B, and PLCG1 were selected for further analysis [23,24,25,26,27]. Co-IP experiments with subsequent WB validated the results in SW480 cells overexpressing MACC1 (SW480/MACC1) and SW620 wild-type cells expressing endogenous MACC1 (Figure 1C).

Further pull-down experiments were conducted with phosphorylated and unphosphorylated versions of the identified peptides to confirm the selectivity and specificity of the interactions. The results demonstrated preferential association of four interactors with phosphorylated compared to the corresponding unphosphorylated peptides: pY365 (STAT5B), pY379 (GRB2, SHP2), and pY789 (SHC1) (Figure 1D). PLCG1 showed a promiscuous binding pattern and was, therefore, excluded from further analysis.

### 3.2. Mutation of pY-Interaction Sites Restricts MACC1-Dependent Functions

**Figure 2:**
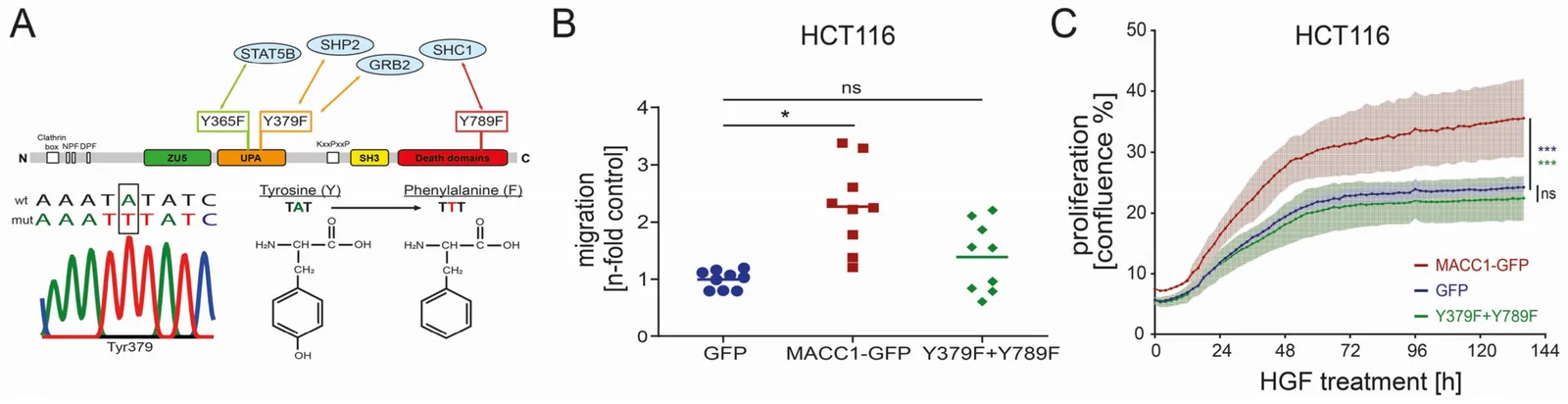
Mutation of interaction sites restricts MACC1-dependent functions. (**A**) Schematic demonstration of site-directed mutagenesis. Combined mutation at two interaction sites (Y379 + Y789) leads to reduced cell migration (**B**) and proliferation (**C**). Results represent means + SD of three independent experiments. Significant results were determined by one-way (**B**) or two-way (**C**) ANOVA and Tukey’s multiple comparison test with a confidence interval of 95% (* = *p* < 0.05, *** = *p* < 0.001). Blue—GFP expressing cells; red—MACC1-GFP expressing cells; green—MACC1-GFP (Y379F + Y789F) expressing cells.

To investigate the functional role of the pY-interaction sites on MACC1, site-directed mutagenesis was employed, targeting two key tyrosine sites, Y379 and Y789 (Figure 2A). These sites mediate interactions with the majority of interactors (GRB2, SHP2, SHC1) found in the MS analysis. These proteins are integral to the regulation of cellular signaling pathways, notably the ERK pathway, which controls critical processes such as cell migration and proliferation [23,24,25,26,27].

To assess the functional impact of masking key tyrosine sites, HCT116 CRC cells transduced either with GFP, MACC1-GFP, or MACC1-GFP carrying the Y379F and Y789F mutations (Y379F + Y789F) were subjected to a Boyden chamber migration assay. Overexpression of MACC1-GFP resulted in a significant increase in cell migration, more than doubling the migration compared to GFP expressing control cells. In contrast, the migratory ability of cells expressing the mutated forms of MACC1 was not significantly different from GFP controls (Figure 2B).

Similarly, the wild-type MACC1-GFP cells exhibited enhanced proliferation under HGF treatment, whereas cells expressing the Y379F + Y789F mutations showed proliferation rates similar to control cells (Figure 2C). To further assess the contribution of the individual tyrosine residues, proliferation was additionally analyzed in cells expressing the Y379F or Y789F single mutants. Mutation of either Y379 or Y789 reduced MACC1-associated proliferation compared with wild-type MACC1-GFP cells, consistent with a functional contribution of both residues (Supplementary Figure S2). Together, these findings support a functional contribution of Y379 and Y789 to MACC1-associated cellular phenotypes, potentially through modulating downstream signaling pathways.

### 3.3. pY-Interaction Sites Contribute to the Signaling Promoting Effect of MACC1

MACC1 is involved in various cellular processes, including the induction of proliferation and cell scattering, particularly in response to HGF stimulation [4]. Additionally, we have shown that MACC1 is directly linked to ERK signaling via phosphorylation by MEK1 and that MACC1 overexpression led to enhanced ERK signaling [13]. This study identifies and confirms MACC1 interactors that are crucial for the signaling mechanisms of RTKs such as MET and EGFR [23,24,25,26,27].

**Figure 3:**
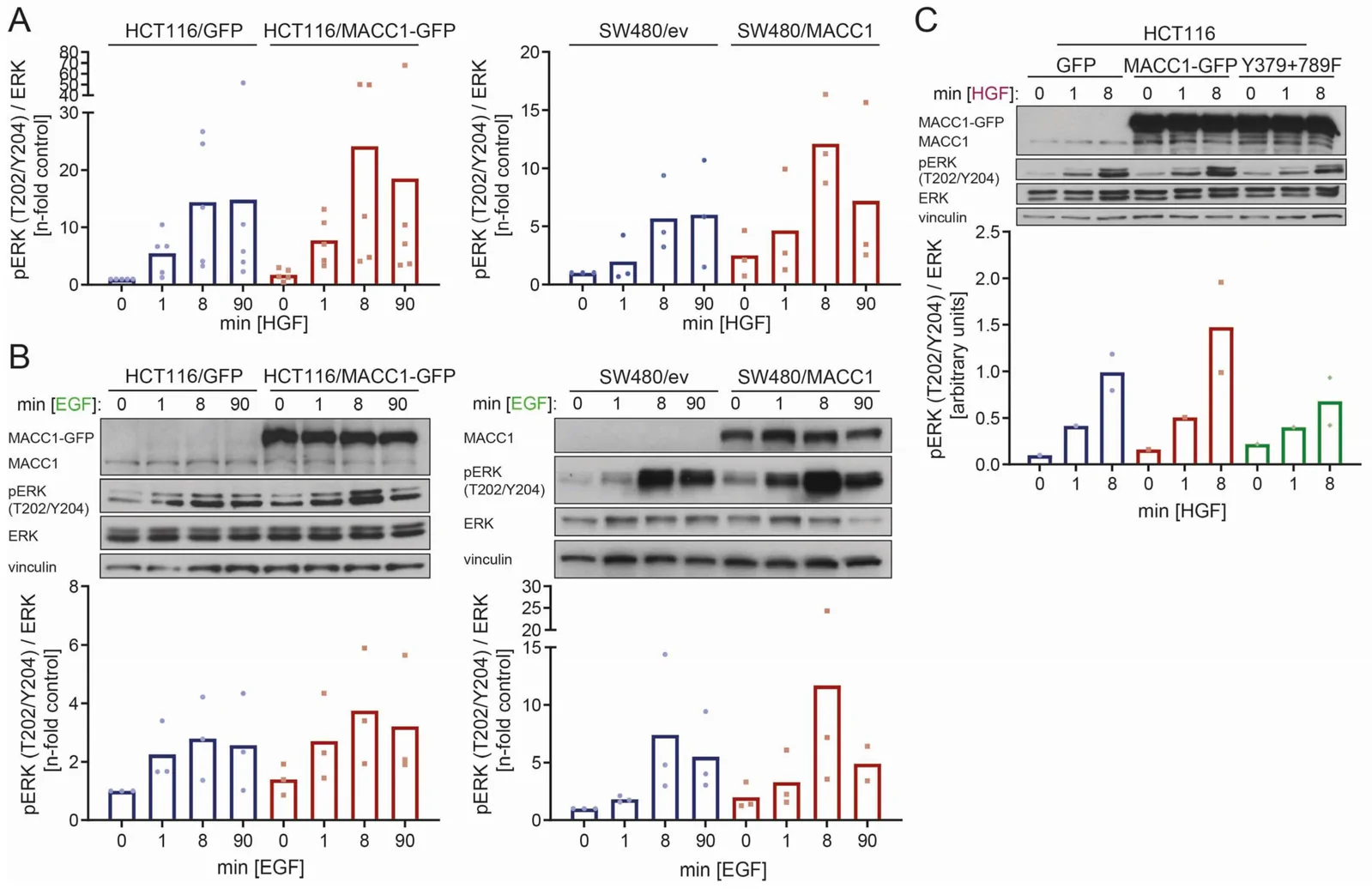
pY-interaction sites are essential for the signaling promoting effect of MACC1. (**A**) In response to HGF treatment, MACC1 overexpression leads to increased ERK activation. Shown here is a newly generated graphical representation of previously published experimental data [13]. (**B**) The same effect is observed with EGF treatment of CRC cells with low vs. high MACC1 expression. Results are demonstrated by WB and respective spot densitometry analysis. Here, the expression of pERK and ERK were normalized to vinculin (loading control) expression before normalizing pERK to ERK, indicating the level of active protein. Values for untreated (0 min) HCT116/GFP or SW480/ev cells were set to 1, resulting in relative phosphorylation levels depicted in the respective bar graphs. Results represent means of at least three independent experiments. (**C**) Mutation of pY-interaction sites (Y379 + Y789) leads to the loss of the signaling promoting effect of MACC1 in response to HGF treatment. Expressions of pERK and ERK were normalized to vinculin (loading control) expression before normalizing pERK to ERK, indicating the level of active protein. Results represent means of two independent experiments. Blue—GFP expressing cells; red—MACC1-GFP expressing cells; green—MACC1-GFP (Y379F + Y789F) expressing cells.

To further explore MACC1’s role in RTK signaling, additional experiments were performed using EGF stimulation. Similar to the HGF stimulation experiments, WB analysis was used to assess ERK activation through phosphorylation at T202 and Y204. Interestingly, SW480 and HCT116 cells overexpressing MACC1 showed stronger phosphorylation of ERK compared to control cells, especially at 1 and 8 min post-stimulation. Elevated pERK baseline levels were also observed in MACC1-overexpressing cells at the 0 min time point, prior to stimulation. After 90 min, ERK activation had mostly plateaued in control cells, while MACC1-high cells showed a decline in pERK levels, suggesting an accelerated and amplified activation loop in the presence of MACC1 overexpression. These results were consistent across the two cell models tested (Figure 3A,B).

To examine the role of specific pY-interaction sites in MACC1-dependent signaling, HCT116 cells expressing MACC1 with tyrosine mutations at Y379 and Y789 (Y379F + Y789F) were subjected to HGF stimulation followed by WB analysis. MACC1 overexpression results in an increase in ERK phosphorylation, while HCT116 cells with the Y379F + Y789F mutations did not exhibit this enhanced phosphorylation, supporting an important role of Y379 and Y789 in MACC1-associated ERK activation (Figure 3C). Of note is that total MACC1-GFP expression was comparable between cells expressing wild-type MACC1-GFP and the Y379F + Y789F mutant (Figure 3C), arguing against differences in MACC1 abundance as the basis for the observed signaling and functional phenotype.

### 3.4. Digital Western Blotting (DigiWest) Reveals a Hyperactivated Signaling Phenotype Exerted by MACC1

**Figure 4:**
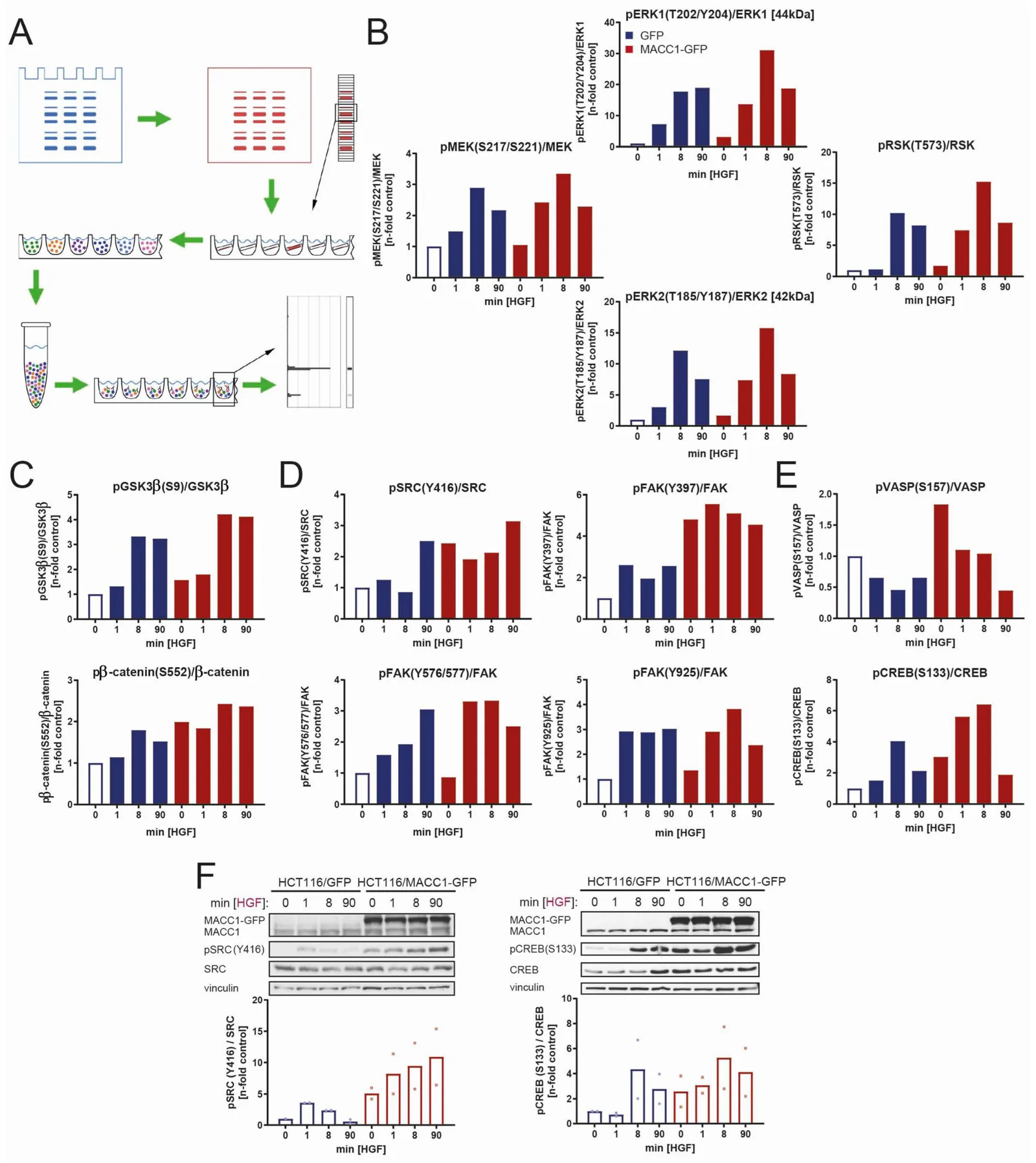
Digital Western blotting reveals a hyperactivated signaling phenotype exerted by MACC1. (**A**) Digital Western blotting (DigiWest) workflow allows the assessment of multiple protein expression patterns from a single WB membrane. Bar graphs illustrate the integrated signal strength of phosphorylated proteins normalized to respective total protein expression, indicating activated or inactivated protein levels. (**B**) MACC1 overexpression in HCT116 cells leads to increased activation of the ERK pathway, represented by elevated phosphorylation of MEK (S217/S221), ERK1 (T202/Y204), ERK2 (T185/Y187) and p-p90 ribosomal s6 kinase (pRSK) (T573). (**C**) Similarly, increased phosphorylation of inactivation site (S9) of GSK3β and activation site (S552) of β-catenin were observed. Further, higher phosphorylation of (**D**) SRC (Y416) and FAK (Y397, Y576/577, Y925) as well as (**E**) VASP (S157) and CREB (S133) was observed in MACC1-overexpressing cells. (**F**) DigiWest findings were validated by standard WB of two selected targets (SRC/pSRC and CREB/pCREB) in two additional biological experiments. The DigiWest and conventional Western blot data were analyzed separately and were not combined for statistical analysis. Expression of respective proteins determined with spot densitometry was normalized to vinculin (loading control) expression before normalizing pSRC to SRC and pCREB to CREB, respectively, indicating the level of active protein. Results in (**F**) represent means of two independent experiments. Blue—GFP expressing cells; red—MACC1-GFP expressing cells.

Cellular signaling pathways often exhibit high levels of crosstalk and redundancy, which complicates the understanding of their individual roles in cellular processes [29,30]. To explore the impact of MACC1 on the cellular signaling landscape, we employed DigiWest, allowing for the assessment of protein expression across a broad range of targets using a single standard WB membrane (Figure 4A). We used the same experimental setup as for standard WB experiments. Figure 4B–E show the (in)activation of various signaling proteins measured by relative phosphorylation of indicated phosphorylation sites: the phosphorylation patterns of MEK (S217/S221), ERK1 (T202/Y204), ERK2 (T185/Y187), and p90 ribosomal S6 kinase (RSK) (T573) reflect a faster and more pronounced activation loop in the ERK signaling pathway through MACC1 overexpression (Figure 4B). This observation aligns with previous findings and serves as a baseline for subsequent data.

Consistently, phosphorylation levels of GSK3β (S9, the inactivated form) and β-catenin (S552, the activated form) were higher in the MACC1-overexpressing cells across all time points. These patterns mirrored the cyclic activation seen with ERK signaling (Figure 4C). Figure 4D shows that the activation of SRC (Y416) and focal adhesion kinase (FAK) (Y397) was enhanced in cells overexpressing MACC1 compared to controls at all time points. Moreover, the subsequent active forms of FAK, namely pFAK (Y576/577) and pFAK (Y925), were more prominently phosphorylated at 1 min and 8 min, respectively. Figure 4E further reveals an increase in activated cAMP response element-binding protein (pCREB, S133) and vasodilator-stimulated phosphoprotein (pVASP, S157) levels in MACC1-high cells from 0 to 8 min, followed by a more pronounced decline at 90 min. Given the exploratory nature of the DigiWest analysis (based on one standard WB, n = 1), selected observations were subsequently evaluated by conventional Western blotting. Increased phosphorylation of SRC and CREB observed in the DigiWest analysis was independently reproduced by conventional Western blot analysis in two additional biological experiments (Figure 4F), underlining the DigiWest findings.

Taken together, the results indicate that MACC1 overexpression accelerates and amplifies the activation of several key signaling molecules (MEK, ERK, RSK, β-catenin, VASP, CREB, FAK, SRC) involved in cellular pathways responsible for cell adhesion, proliferation, survival, and migration as well as tumor progression and metastasis [13,31,32,33,34,35,36,37]. MACC1 overexpression leads to faster activation loops, with already elevated baseline levels of active signaling proteins.

### 3.5. Pharmacological Inhibition Reveals Vulnerabilities in MACC1-Associated Signaling

**Figure 5:**
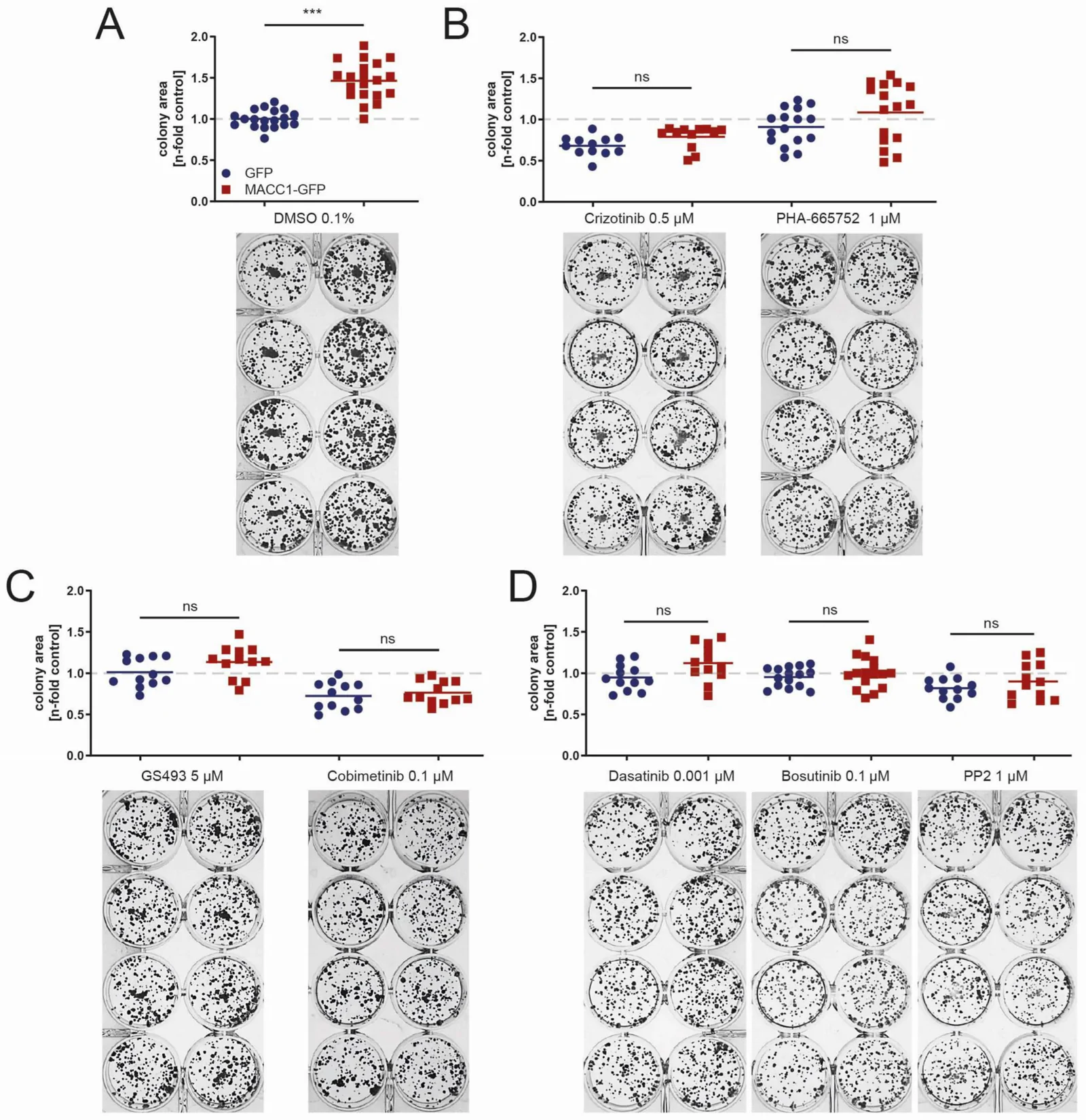
Pharmacological inhibition reveals vulnerabilities in MACC1-associated signaling. The clonogenic assay was used to test the inhibitory potential of several drugs on MACC1-dependent cell function in HCT116/GFP and HCT116/MACC1-GFP cells. Colony formation was significantly increased by MACC1 overexpression (**A**), which could be reduced to control levels with inhibitors of (**B**) MET (critzotinib, PHA-665752), (**C**) SHP2 (GS493), MEK (cobimetinib), or (**D**) SRC (dasatinib, bosutinib, PP2). Colony covered area was quantified using the Colony Area plugin in ImageJ and normalized to values of solvent-treated HCT116/GFP cells (blue dashed line), resulting in relative reproductive viability depicted in the dot plots. Results represent means of at least three independent experiments with one representative example illustrated below. Significant results were determined by two-way ANOVA with Sidak’s post hoc test with a confidence interval of 95% (*** = *p* < 0.001).

The landscape of cancer treatment has evolved with the addition of targeted therapies, often focusing on inhibitors of receptor tyrosine kinase (RTK) signaling and downstream pathways [38,39]. To investigate whether the MACC1-associated signaling phenotype identified in this study is pharmacologically vulnerable, we assessed the effects of various inhibitors on MACC1-induced cellular functions. Clonogenic assays were performed with HCT116/GFP and HCT116/MACC1-GFP cells using DMSO (control) or inhibitors targeting MET (crizotinib, PHA-665752), SHP2 (GS493), MEK (cobimetinib), and SRC-associated signaling (dasatinib, bosutinib, PP2). MACC1 overexpression significantly increased colony formation compared to the control cells (Figure 5A). Treatment with the tested inhibitors reduced the enhanced colony-forming capacity of MACC1-overexpressing cells toward levels observed in the corresponding GFP control cells (Figure 5B–D). Within-genotype comparisons with the respective vehicle controls showed that this reduction was significant for crizotinib, cobimetinib, bosutinib, and PP2 in HCT116/MACC1-GFP cells. In contrast, only crizotinib significantly reduced colony formation in HCT116/GFP control cells compared with vehicle treatment (Supplementary Figure S3). These findings indicate an increased pharmacological sensitivity of the MACC1-overexpressing phenotype to the inhibition of selected components of the MACC1-associated signaling network. Notably, this sensitivity to interference with SRC-associated signaling was observed with two of the three tested inhibitors, bosutinib and PP2, supporting the robustness of this pharmacological phenotype across different compounds.

## 4. Discussion

In this study, we identified and validated several key interaction partners of MACC1 through mass spectrometry (MS) and co-immunoprecipitation (Co-IP). These interactors involved in RTK signalosomes preferentially associated with distinct phosphorylated MACC1-derived peptides: pY365 (STAT5B), pY379 (GRB2 and SHP2), and pY789 (SHC1). Mutations at two of these critical tyrosine sites (Y379F + Y789F) reduced MACC1-induced activation of ERK, migration, and proliferation in CRC cells, identifying these sites as potential drug intervention points. Additionally, we demonstrated that MACC1 promotes ERK signaling in response to both HGF and EGF stimulation, suggesting that MACC1 mediates malignant signaling and subsequent effects (e.g., increased cell proliferation and motility) below different RTKs. These effects are independent of MET overexpression alone. Using DigiWest, we expanded our understanding of the MACC1 signaling network. Inhibiting central mediators of this network effectively limited MACC1-driven colony formation, highlighting potential therapeutic targets for MACC1-stratified high-risk cancer patients or combinatorial treatment.

MACC1 phosphorylation at Y379 and Y789 apparently plays a significant role in regulating cellular processes in colorectal cancer (CRC), a phenomenon observed in various studies.

All identified MACC1 interactors are involved in key cellular functions, including malignant processes: SHP2, in association with GAB1, mediates cell migration, cytoskeletal reorganization, and organ development through ERK signaling [24,25,40,41,42]. SHP2 mutations are associated with various cancers, including leukemia and tumors of the colon, breast, and lung, and inhibited SHP2 binding through the mutation of these sites led to a reduction in anchorage-independent growth of breast cancer cells [43]. GRB2 and SHC1, as central adaptor proteins, modulate signaling by connecting downstream effectors such as SOS, RAS, or GAB1 to RTKs. Abnormal function or expression is directly linked to tumor progression or metastasis formation in various entities [44].

In addition, the phosphorylation of GAB1 at different tyrosine sites is crucial for the activation of downstream signaling (e.g., ERK)—predominantly mediated via GAB1-SHP2 binding—in response to multiple stimuli such as HGF, EGF, or VEGF. Mutations of these sites impair GAB1’s ability to mediate biological functions, such as cell migration, cytoskeletal rearrangement, and organ development [24,25,40,41,42]. Similarly, phosphorylated tyrosines on β4-integrin are essential for SHP2 binding and downstream signaling via a SHP2-SRC-GAB1-GRB2-ERK axis.

Based on these interactor functions and our findings that MACC1 overexpression enhances ERK activation in CRC cells, we sought to further explore the MACC1 signaling landscape by using DigiWest. We found the upregulation of the phosphorylation of several proteins, which are involved in key signaling pathways, highlighting MACC1’s role in regulating their activity.

We observed the hyperactivation of the ERK pathway through increased phosphorylation of MEK, ERK1/2, and RSK. This aligns with earlier studies demonstrating that MACC1 induces tumorigenic effects via ERK signaling [13,33,34]. MACC1 has already been found to induce target gene expression (e.g., MYC, cyclin D1/E, and MMPs) or chemoresistance and stemness features via (AKT/)β-catenin-signaling [5,6]. Moreover, we have demonstrated a MACC1–β-catenin–S100A4 signaling axis inducing metastasis in vitro and in vivo [35]. This study is partially based on our DigiWest findings showing MACC1-induced β-catenin activation through the phosphorylation of GSK3β at S9 and β-catenin at S552 (Figure 4C). Both phosphorylations are directly regulated by AKT and PKA [45,46,47]. Thus, downstream mediators of PKA including CREB (pS133) and VASP (pS157) demonstrated higher activation patterns upon MACC1 overexpression. VASP, involved in cytoskeletal dynamics, governs cell adhesion, polarity, and motility [36]. The overexpression and increased activity of CREB, a transcription factor linked to proliferation and migration, have been associated with tumor progression and metastasis [37].

MACC1 overexpression also conferred hyperactivation to FAK (Y397, Y576/577 and Y925) and SRC (Y416), with Y397 as the binding site for SRC. The sequential phosphorylation of FAK at the other sites transfers activation to PI3K and ERK pathways responsible for cell survival, invasion, and migration [32]. Taken together, these findings offer new perspectives on MACC1’s influence in cancer progression and metastasis, emphasizing its pivotal role in fostering a metastatic phenotype.

Our findings highlight recurring phosphorylation/activation patterns in MACC1-associated signaling. These feedback loops, common in signaling pathways, regulate signal duration and strength. For instance, ERK activation suppresses its upstream regulators, controlling its own activity [48]. Minor changes in signaling cascades can significantly affect cell fate: for example, the strength and duration of ERK activation influence transcriptional responses and gene expression patterns [49,50]. More interestingly, related ligands FGF-7 and FGF-10 guide distinct cellular functions (proliferation or migration) through different signaling timeframes dependent on the recruitment of SH3 domain-binding protein 4 (SH3BP4) to FGFR [51]—SH3BP4 shares nearly 50% sequence homology with MACC1 [4].

Beyond self-regulatory mechanisms, signaling pathways adjust and crosstalk dynamically. A key example is the (multi)adaptor protein GAB1, which amplifies signaling from different RTKs by recruiting downstream effectors like PI3K and the GRB2-SOS-RAS complex, thereby also modulating the balance between PI3K and RAS-ERK signaling. All RTKs typically engage a specific set of adaptors and mediators, which often overlap significantly. The resulting signaling response varies depending on the recruitment partners and their patterns of interaction [48]. Our findings, showing that MACC1 facilitates ERK signaling in response to HGF as well as EGF, support this notion and suggest MACC1’s role as a malignant mediator of signaling below various RTKs. 

Especially in cancer, RTKs such as MET undergo ligand-independent activation as a consequence of overexpression. This phenomenon could explain the higher baseline activation of most investigated target proteins like ERK, SRC, and CREB in unstimulated (0 min) MACC1-high conditions. However, this (Supplementary Figure S1; MET) and our previous study [52] (EGFR) show that neither MET nor EGFR demonstrate this higher baseline activation under these conditions. Additionally, abrogating the increased signaling effect through the mutation of pY-interaction sites emphasizes the malignant mediator role of MACC1 within downstream signaling networks besides its effects on MET expression. Also, the fact that inhibiting several targets within the MACC1 signaling landscape (i.e., MET, MEK, SRC, SHP2) reduced MACC1-specific colony formation to the control cell level underlines this role.

Besides sharing similar signaling proteins which MACC1 interacts with, RTK transactivation cannot be excluded as a mechanism for MACC1 being a multi-RTK enhancer. In this context, SRC is a very interesting player: SRC is a key signaling molecule that operates downstream of several receptor tyrosine kinases (RTKs) such as MET, EGFR, and PDGFR. In addition, SRC can directly phosphorylate these RTKs, activating them in the process [32,53]. Research by Song et al. revealed that SRC plays a pivotal role in coordinating the signaling pathways of MET, EGFR, and IGFR in CRC cells [54]. SRC also influences GAB1 signaling, both in a MET-dependent and independent manner [55,56]. Interestingly, studies have shown that SHP2 activates SRC as part of its functional mechanism [43,57,58,59] and identified SRC as a key driver of breast cancer progression in a PKA-dependent manner in vivo [31].

Based on these findings, we propose novel MACC1/SHP2/SRC/ERK and MACC1/PKA/SRC/CREB signaling axes that promote malignancy in response to different growth factor signals. These axes represent a new connection between RTK and PKA signaling, a pathway that has not been extensively studied [60]. Overall, our data suggest that MACC1 rewires cellular signaling networks to favor tumorigenic and metastatic pathways.

To assess the functional impact of MACC1-dependent signaling and explore potential therapeutic targets, we performed clonogenic assays with various inhibitors. These included inhibitors targeting MET (crizotinib, PHA-665752), SHP2 (GS493), MEK (cobimetinib), and SRC (dasatinib, bosutinib, PP2). All treatments effectively reduced MACC1-driven colony formation to baseline levels. Notably, several of these inhibitors have already been tested or approved for clinical use (e.g., crizotinib, cobimetinib, dasatinib, bosutinib), suggesting their potential for repurposing [61] to target MACC1-associated pathways in cancer therapy. We will further assess MACC1’s role as a malignant adaptor within RTK signalosomes, as it shares features with other key players of RTK signaling, e.g., the (multi)adaptor protein GAB1, as described above. Similarly, structural and functional similarities between MACC1 and SH3BP4 have been uncovered by us [4,52] and others [51]. Here, we want to follow up on developing novel intracellular antibodies or find mimicking inhibitors via high-throughput drug screening that block the MACC1 interaction sites (Y379 and Y789) [62]. By this, we hope to fully exploit the potential of MACC1 as a multifaceted biomarker and therapeutic target.

## 5. Limitations

While our findings support a phosphorylation-dependent adaptor function of MACC1 in enhancing downstream RTK signaling, several limitations should be acknowledged. The mechanistic analyses were primarily performed in colorectal cancer cell models, and the proposed signaling architecture requires further validation in vivo and in patient-derived material. Although phospho-peptide pull-down experiments demonstrate preferential association of GRB2, SHP2, SHC1, and STAT5B with distinct phosphorylated MACC1-derived peptides, and cellular Co-IP supports their association with full-length MACC1, including at endogenous MACC1 levels in SW620 cells, these experiments do not formally establish direct, residue-specific binding. Further biochemical and structural studies will therefore be required to define the molecular basis, binding affinities, and spatiotemporal dynamics of these interactions.

Furthermore, while analysis of the individual Y379F and Y789F mutants supports a contribution of both residues to MACC1-associated proliferation, their individual contributions to migration and ERK signaling were not assessed. The phosphorylation of Y379 and Y789 in full-length cellular MACC1 was also not directly demonstrated. Comparable expression of wild-type and Y379F + Y789F MACC1-GFP argues against reduced protein abundance as an explanation for the observed signaling phenotype; however, the effects of the substitutions on MACC1 localization, stability, or protein conformation cannot be formally excluded.

Finally, the pharmacological experiments identify vulnerabilities associated with the MACC1-overexpressing phenotype but do not establish target-specific dependencies. While matched GFP control cells indicate that the effects of most inhibitors cannot be explained solely by a general suppression of colony formation, the clonogenic assay does not distinguish cytostatic from cytotoxic effects. Moreover, dose–response relationships and direct target engagement were not assessed, the compounds may exhibit off-target activities, and genetic validation of the respective signaling components was not performed. Further studies using endogenous MACC1 manipulation, additional RTK systems, and patient-derived or in vivo models will therefore be required to establish the molecular targets underlying these pharmacological sensitivities and to determine whether MACC1 similarly modulates signaling downstream of additional RTKs beyond HGF/MET and EGF/EGFR.

## 6. Conclusions

We identify a phosphorylation-dependent adaptor function of MACC1 associated with the hyperactivation of downstream RTK signaling and interactions with key signaling mediators, including GRB2, SHP2, SHC1, and STAT5B. Enhanced signaling in response to both HGF and EGF, together with the activation of multiple oncogenic signaling pathways, suggests a broader role of MACC1 in modulating RTK-associated signaling. MACC1 thereby promotes malignant phenotypes associated with colorectal cancer metastasis and reveals pharmacological vulnerabilities to clinically relevant signaling inhibitors. These findings provide a mechanistic framework for the signaling promoting activity of MACC1 and support further investigation of its role as a modulator of RTK-associated signaling and as a potential therapeutic vulnerability in metastasis-prone colorectal cancer.

## Data Availability

The original contributions presented in this study are included in the article/Supplementary Material. Further inquiries can be directed to the corresponding author.

## Supplementary Materials

The following supporting information can be downloaded at: https://www.mdpi.com/article/10.3390/biom16091267/s1. Figure S1: MACC1 overexpression enhances ERK but not MET activation; Figure S2: Individual mutation of Y379 and Y789 reduces MACC1-associated proliferation; Figure S3: Effect of pharmacological inhibitors on colony formation within GFP control and MACC1-GFP cells. Table S1: List of antibodies used in standard and digital Western Blotting. The original Western blot images can be found in the Supplementary Materials.

## References

[1] Gerstberger S., Jiang Q., Ganesh K. (2023). Metastasis. Cell.

[2] Passaro A., Al Bakir M., Hamilton E.G., Diehn M., André F., Roy-Chowdhuri S., Mountzios G., Witsuba I., Swanton C., Peters S. (2024). Cancer Biomarkers—Emerging Trends and Clinical Implications for Personalized Treatment. Cell.

[3] Esposito M., Ganesan S., Kang Y. (2021). Emerging Strategies for Treating Metastasis. Nat. Cancer.

[4] Stein U., Walther W., Arlt F., Schwabe H., Smith J., Fichtner I., Birchmeier W., Schlag P.M. (2009). MACC1, a Newly Identified Key Regulator of HGF-MET Signaling, Predicts Colon Cancer Metastasis. Nat. Med..

[5] Radhakrishnan H., Walther W., Zincke F., Kobelt D., Imbastari F., Erdem M., Kortüm B., Dahlmann M., Stein U. (2018). MACC1-the First Decade of a Key Metastasis Molecule from Gene Discovery to Clinical Translation. Cancer Metastasis Rev..

[6] Schöpe P.C., Torke S., Kobelt D., Kortüm B., Treese C., Dumbani M., Güllü N., Walther W., Stein U. (2024). MACC1 Revisited—An in-Depth Review of a Master of Metastasis. Biomark. Res..

[7] Kurochkina N., Guha U. (2013). SH3 Domains: Modules of Protein-Protein Interactions. Biophys. Rev..

[8] Wang C., Yu C., Ye F., Wei Z., Zhang M. (2012). Structure of the ZU5-ZU5-UPA-DD Tandem of Ankyrin-B Reveals Interaction Surfaces Necessary for Ankyrin Function. Proc. Natl. Acad. Sci. USA.

[9] Park H.H., Lo Y.-C., Lin S.-C., Wang L., Yang J.K., Wu H. (2007). The Death Domain Superfamily in Intracellular Signaling of Apoptosis and Inflammation. Annu. Rev. Immunol..

[10] Felder S., Zhou M., Hu P., Ureña J., Ullrich A., Chaudhuri M., White M., Shoelson S.E., Schlessinger J. (1993). SH2 Domains Exhibit High-Affinity Binding to Tyrosine-Phosphorylated Peptides yet Also Exhibit Rapid Dissociation and Exchange. Mol. Cell. Biol..

[11] Filippakopoulos P., Müller S., Knapp S. (2009). SH2 Domains: Modulators of Nonreceptor Tyrosine Kinase Activity. Curr. Opin. Struct. Biol..

[12] Cohen P. (2002). The Origins of Protein Phosphorylation. Nat. Cell Biol..

[13] Kobelt D., Perez-Hernandez D., Fleuter C., Dahlmann M., Zincke F., Smith J., Migotti R., Popp O., Burock S., Walther W. (2021). The Newly Identified MEK1 Tyrosine Phosphorylation Target MACC1 Is Druggable by Approved MEK1 Inhibitors to Restrict Colorectal Cancer Metastasis. Oncogene.

[14] Treindl F., Ruprecht B., Beiter Y., Schultz S., Döttinger A., Staebler A., Joos T.O., Kling S., Poetz O., Fehm T. (2016). A Bead-Based Western for High-Throughput Cellular Signal Transduction Analyses. Nat. Commun..

[15] Rafehi H., Orlowski C., Georgiadis G.T., Ververis K., El-Osta A., Karagiannis T.C. (2011). Clonogenic Assay: Adherent Cells. J. Vis. Exp. JoVE.

[16] Puck T.T., Marcus P.I. (1956). Action of X-Rays on Mammalian Cells. J. Exp. Med..

[17] Franken N.A.P., Rodermond H.M., Stap J., Haveman J., van Bree C. (2006). Clonogenic Assay of Cells In Vitro. Nat. Protoc..

[18] Médard G., Pachl F., Ruprecht B., Klaeger S., Heinzlmeir S., Helm D., Qiao H., Ku X., Wilhelm M., Kuehne T. (2015). Optimized Chemical Proteomics Assay for Kinase Inhibitor Profiling. J. Proteome Res..

[19] Cox J., Neuhauser N., Michalski A., Scheltema R.A., Olsen J.V., Mann M. (2011). Andromeda: A Peptide Search Engine Integrated into the MaxQuant Environment. J. Proteome Res..

[20] Cox J., Mann M. (2008). MaxQuant Enables High Peptide Identification Rates, Individualized p.p.b.-Range Mass Accuracies and Proteome-Wide Protein Quantification. Nat. Biotechnol..

[21] Fagerberg L., Hallström B.M., Oksvold P., Kampf C., Djureinovic D., Odeberg J., Habuka M., Tahmasebpoor S., Danielsson A., Edlund K. (2014). Analysis of the Human Tissue-Specific Expression by Genome-Wide Integration of Transcriptomics and Antibody-Based Proteomics. Mol. Cell. Proteom..

[22] Uhlén M., Fagerberg L., Hallström B.M., Lindskog C., Oksvold P., Mardinoglu A., Sivertsson Å., Kampf C., Sjöstedt E., Asplund A. (2015). Tissue-Based Map of the Human Proteome. Science.

[23] Boccaccio C., Comoglio P.M. (2006). Invasive Growth: A MET-Driven Genetic Programme for Cancer and Stem Cells. Nat. Rev. Cancer.

[24] Cunnick J.M., Meng S., Ren Y., Desponts C., Wang H.-G., Djeu J.Y., Wu J. (2002). Regulation of the Mitogen-Activated Protein Kinase Signaling Pathway by SHP2. J. Biol. Chem..

[25] Schaeper U., Gehring N.H., Fuchs K.P., Sachs M., Kempkes B., Birchmeier W. (2000). Coupling of Gab1 to C-Met, Grb2, and Shp2 Mediates Biological Responses. J. Cell Biol..

[26] Wee P., Wang Z. (2017). Epidermal Growth Factor Receptor Cell Proliferation Signaling Pathways. Cancers.

[27] Scaltriti M., Baselga J. (2006). The Epidermal Growth Factor Receptor Pathway: A Model for Targeted Therapy. Clin. Cancer Res..

[28] Blom N., Gammeltoft S., Brunak S. (1999). Sequence and Structure-Based Prediction of Eukaryotic Protein Phosphorylation Sites1. J. Mol. Biol..

[29] Vogelstein B., Kinzler K.W. (2004). Cancer Genes and the Pathways They Control. Nat. Med..

[30] Sever R., Brugge J.S. (2015). Signal Transduction in Cancer. Cold Spring Harb. Perspect. Med..

[31] Beristain A.G., Molyneux S.D., Joshi P.A., Pomroy N.C., Di Grappa M.A., Chang M.C., Kirschner L.S., Privé G.G., Pujana M.A., Khokha R. (2015). PKA Signaling Drives Mammary Tumorigenesis through Src. Oncogene.

[32] Sulzmaier F.J., Jean C., Schlaepfer D.D. (2014). FAK in Cancer: Mechanistic Findings and Clinical Applications. Nat. Rev. Cancer.

[33] Zhang R., Shi H., Chen Z., Wu Q., Ren F., Huang H. (2011). Effects of Metastasis-Associated in Colon Cancer 1 Inhibition by Small Hairpin RNA on Ovarian Carcinoma OVCAR-3 Cells. J. Exp. Clin. Cancer Res. CR.

[34] Wang G., Kang M.-X., Lu W.-J., Chen Y., Zhang B., Wu Y.-L. (2012). MACC1: A Potential Molecule Associated with Pancreatic Cancer Metastasis and Chemoresistance. Oncol. Lett..

[35] Kortüm B., Radhakrishnan H., Zincke F., Sachse C., Burock S., Keilholz U., Dahlmann M., Walther W., Dittmar G., Kobelt D. (2022). Combinatorial Treatment with Statins and Niclosamide Prevents CRC Dissemination by Unhinging the MACC1-β-Catenin-S100A4 Axis of Metastasis. Oncogene.

[36] Döppler H., Storz P. (2013). Regulation of VASP by Phosphorylation. Cell Adhes. Migr..

[37] Steven A., Seliger B. (2016). Control of CREB Expression in Tumors: From Molecular Mechanisms and Signal Transduction Pathways to Therapeutic Target. Oncotarget.

[38] Bhullar K.S., Lagarón N.O., McGowan E.M., Parmar I., Jha A., Hubbard B.P., Rupasinghe H.P.V. (2018). Kinase-Targeted Cancer Therapies: Progress, Challenges and Future Directions. Mol. Cancer.

[39] Punt C.J.A., Koopman M., Vermeulen L. (2017). From Tumour Heterogeneity to Advances in Precision Treatment of Colorectal Cancer. Nat. Rev. Clin. Oncol..

[40] Cunnick J.M., Mei L., Doupnik C.A., Wu J. (2001). Phosphotyrosines 627 and 659 of Gab1 Constitute a Bisphosphoryl Tyrosine-Based Activation Motif (BTAM) Conferring Binding and Activation of SHP2. J. Biol. Chem..

[41] Schaeper U., Vogel R., Chmielowiec J., Huelsken J., Rosario M., Birchmeier W. (2007). Distinct Requirements for Gab1 in Met and EGF Receptor Signaling In Vivo. Proc. Natl. Acad. Sci. USA.

[42] Laramé M., Chabot C., Cloutier M., Stenne R., Holgado-Madruga M., Wong A.J., Royal I. (2007). The Scaffolding Adapter Gab1 Mediates Vascular Endothelial Growth Factor Signaling and Is Required for Endothelial Cell Migration and Capillary Formation. J. Biol. Chem..

[43] Bertotti A., Comoglio P.M., Trusolino L. (2006). Β4 Integrin Activates a Shp2–Src Signaling Pathway That Sustains HGF-Induced Anchorage-Independent Growth. J. Cell Biol..

[44] Giubellino A., Arany P.R., Wu W.-S., Hu C.-T. (2010). Grb2 and Other Adaptor Proteins in Tumor Metastasis. Signal Transduction in Cancer Metastasis.

[45] Fang D., Hawke D., Zheng Y., Xia Y., Meisenhelder J., Nika H., Mills G.B., Kobayashi R., Hunter T., Lu Z. (2007). Phosphorylation of Beta-Catenin by AKT Promotes Beta-Catenin Transcriptional Activity. J. Biol. Chem..

[46] Zhou X., Wang H., Burg M.B., Ferraris J.D. (2013). Inhibitory Phosphorylation of GSK-3β by AKT, PKA, and PI3K Contributes to High NaCl-Induced Activation of the Transcription Factor NFAT5 (TonEBP/OREBP). Am. J. Physiol. Ren. Physiol..

[47] Taurin S., Sandbo N., Qin Y., Browning D., Dulin N.O. (2006). Phosphorylation of Beta-Catenin by Cyclic AMP-Dependent Protein Kinase. J. Biol. Chem..

[48] Kolch W., Halasz M., Granovskaya M., Kholodenko B.N. (2015). The Dynamic Control of Signal Transduction Networks in Cancer Cells. Nat. Rev. Cancer.

[49] Murphy L.O., Smith S., Chen R.-H., Fingar D.C., Blenis J. (2002). Molecular Interpretation of ERK Signal Duration by Immediate Early Gene Products. Nat. Cell Biol..

[50] Sarkisian C.J., Keister B.A., Stairs D.B., Boxer R.B., Moody S.E., Chodosh L.A. (2007). Dose-Dependent Oncogene-Induced Senescence In Vivo and Its Evasion during Mammary Tumorigenesis. Nat. Cell Biol..

[51] Francavilla C., Rigbolt K.T.G., Emdal K.B., Carraro G., Vernet E., Bekker-Jensen D.B., Streicher W., Wikström M., Sundström M., Bellusci S. (2013). Functional Proteomics Defines the Molecular Switch Underlying FGF Receptor Trafficking and Cellular Outputs. Mol. Cell.

[52] Imbastari F., Dahlmann M., Sporbert A., Mattioli C.C., Mari T., Scholz F., Timm L., Twamley S., Migotti R., Walther W. (2021). MACC1 Regulates Clathrin-Mediated Endocytosis and Receptor Recycling of Transferrin Receptor and EGFR in Colorectal Cancer. Cell. Mol. Life Sci. CMLS.

[53] Bromann P.A., Korkaya H., Courtneidge S.A. (2004). The Interplay between Src Family Kinases and Receptor Tyrosine Kinases. Oncogene.

[54] Song N., Qu X., Liu S., Zhang S., Liu J., Qu J., Zheng H., Liu Y., Che X. (2017). Dual Inhibition of MET and SRC Kinase Activity as a Combined Targeting Strategy for Colon Cancer. Exp. Ther. Med..

[55] Chan P.-C., Chen Y.-L., Cheng C.-H., Yu K.-C., Cary L.A., Shu K.-H., Ho W.L., Chen H.-C. (2003). Src Phosphorylates Grb2-Associated Binder 1 upon Hepatocyte Growth Factor Stimulation. J. Biol. Chem..

[56] Chan P.-C., Sudhakar J.N., Lai C.-C., Chen H.-C. (2010). Differential Phosphorylation of the Docking Protein Gab1 by C-Src and the Hepatocyte Growth Factor Receptor Regulates Different Aspects of Cell Functions. Oncogene.

[57] Ren Y., Meng S., Mei L., Zhao Z.J., Jove R., Wu J. (2004). Roles of Gab1 and SHP2 in Paxillin Tyrosine Dephosphorylation and Src Activation in Response to Epidermal Growth Factor. J. Biol. Chem..

[58] Yu D.H., Qu C.K., Henegariu O., Lu X., Feng G.S. (1998). Protein-Tyrosine Phosphatase Shp-2 Regulates Cell Spreading, Migration, and Focal Adhesion. J. Biol. Chem..

[59] Zhang S.Q., Yang W., Kontaridis M.I., Bivona T.G., Wen G., Araki T., Luo J., Thompson J.A., Schraven B.L., Philips M.R. (2004). Shp2 Regulates SRC Family Kinase Activity and Ras/Erk Activation by Controlling Csk Recruitment. Mol. Cell.

[60] Caldwell G.B., Howe A.K., Nickl C.K., Dostmann W.R., Ballif B.A., Deming P.B. (2012). Direct Modulation of the Protein Kinase A Catalytic Subunit α by Growth Factor Receptor Tyrosine Kinases. J. Cell. Biochem..

[61] Pushpakom S., Iorio F., Eyers P.A., Escott K.J., Hopper S., Wells A., Doig A., Guilliams T., Latimer J., McNamee C. (2019). Drug Repurposing: Progress, Challenges and Recommendations. Nat. Rev. Drug Discov..

[62] Rabbitts T.H. (2023). Intracellular Antibodies for Drug Discovery and as Drugs of the Future. Antibodies.

