# Supplementary material for: MACC1 Hyperactivates Receptor Tyrosine Kinase Signaling Through Phosphorylation-Dependent Adaptor Activity in Colorectal Cancer Cells
Source: Biomolecules. 2026 Sep 2;16(9):1267. doi: 10.3390/biom16091267 (PMC13604596; doi:10.3390/biom16091267)
Supplement: Supplementary file 1 [file biomolecules-16-01267-s001.zip › biomolecules-4502079-supplementary.pdf]

Supplementary Materials

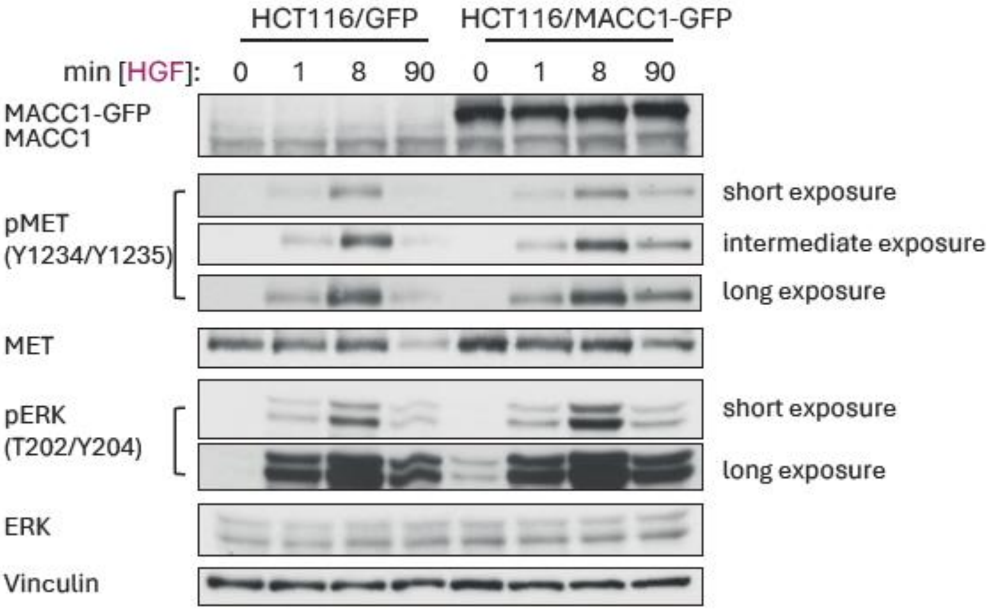

Supplementary Figure S1: *MACC1 overexpression enhances ERK but not MET activation. In response to HGF treatment as well as in untreated conditions, MACC1 overexpression leads to increased ERK phosphorylation compared to control cells. The effect in untreated cells is not observed for activating MET phosphorylations.*

analyte	dilution	antibody	product number/company
**Standard WB**			
MACC1	1 : 1 000	rabbit, polyclonal	HPA020081 / Sigma-Aldrich
ERK	1 : 1 000	rabbit, polyclonal	#9102 / Cell Signaling
pERK (T202/Y204)	1 : 1 000	rabbit, polyclonal	#9101 / Cell Signaling
MET	1 : 1 000	rabbit, monoclonal	#8198 / Cell Signaling
pMET (Y1234/1235)	1 : 1 000	rabbit, monoclonal	#3077 / Cell Signaling
CREB	1 : 1 000	rabbit, monoclonal	#9197 / Cell Signaling
pCREB (S133)	1 : 1 000	rabbit, monoclonal	#9198 / Cell Signaling
SRC	1 : 2 000	rabbit, monoclonal	#2109 / Cell Signaling
pSRC (Y416)	1 : 1 000	rabbit, polyclonal	#2101 / Cell Signaling
β-actin	1 : 25 000	mouse, monoclonal	A1978 / Sigma-Aldrich
vinculin	1 : 2 000	mouse, monoclonal	V9131 / Sigma-Aldrich
PLCG1	1 : 2 000	rabbit, polyclonal	sc-81 / Santa Cruz Biotech
STAT5B	1 : 1 000	mouse, monoclonal	sc-1656 / Santa Cruz Biotech
SHP2	1 : 1 000	rabbit, polyclonal	sc-280 / Santa Cruz Biotech
SHC	1 : 1 000	rabbit, polyclonal	sc-1695 / Santa Cruz Biotech
GRB2	1 : 1 000	rabbit, polyclonal	sc-255 / Santa Cruz Biotech
Anti-rabbit-HRP	1 : 20 000	goat, polyclonal	W4011 / Promega
Anti-mouse-HRP	1 : 20 000	goat, polyclonal	# 31430 / Thermo Fisher
**DigiWest**			
β-catenin	1 : 200	rabbit, polyclonal	06-734 / Merck-Millipore
pβ-catenin (S552)	1 : 200	rabbit, polyclonal	#9566 / Cell Signaling
CREB	1 : 200	rabbit, monoclonal	#9197 / Cell Signaling
pCREB (S133)	1 : 1 000	rabbit, monoclonal	#9198 / Cell Signaling
ERK1/2	1 : 200	rabbit, monoclonal	#4695 / Cell Signaling
pERK1/2 (T202/Y204)	1 : 2 000	rabbit, monoclonal	#4370 / Cell Signaling
FAK	1 : 200	rabbit, monoclonal	2146-1 / Epitomics
pFAK (Y397)	1 : 200	rabbit, monoclonal	ab81298 / abcam
pFAK (Y576/Y577)	1 : 200	rabbit, monoclonal	2183-1 / Epitomics
pFAK (Y925)	1 : 200	rabbit, polyclonal	ab38512 / abcam
GSK3β	1 : 200	rabbit, monoclonal	#9315 / Cell Signaling
pGSK3β (S9)	1 : 200	rabbit, polyclonal	#9336 / Cell Signaling
MACC1	1 : 200	rabbit, polyclonal	HPA020081 / Sigma-Aldrich
MEK1	1 : 200	rabbit, polyclonal	#9124 / Cell Signaling
pMEK1/2 (S217/S221)	1 : 200	rabbit, monoclonal	#9154 / Cell Signaling
RSK1	1 : 200	rabbit, polyclonal	06-668 / Merck-Millipore
pRSK1 (T573)	1 : 200	rabbit, monoclonal	ab62324 / abcam
SRC	1 : 200	rabbit, monoclonal	#2109 / Cell Signaling
pSRC (Y416)	1 : 200	rabbit, monoclonal	#6943 / Cell Signaling
VASP	1 : 200	rabbit, monoclonal	#3132 / Cell Signaling
pVASP (S157)	1 : 200	rabbit, polyclonal	#3111 / Cell Signaling

Supplementary Table S1: List of antibodies used in standard and digital Western Blotting

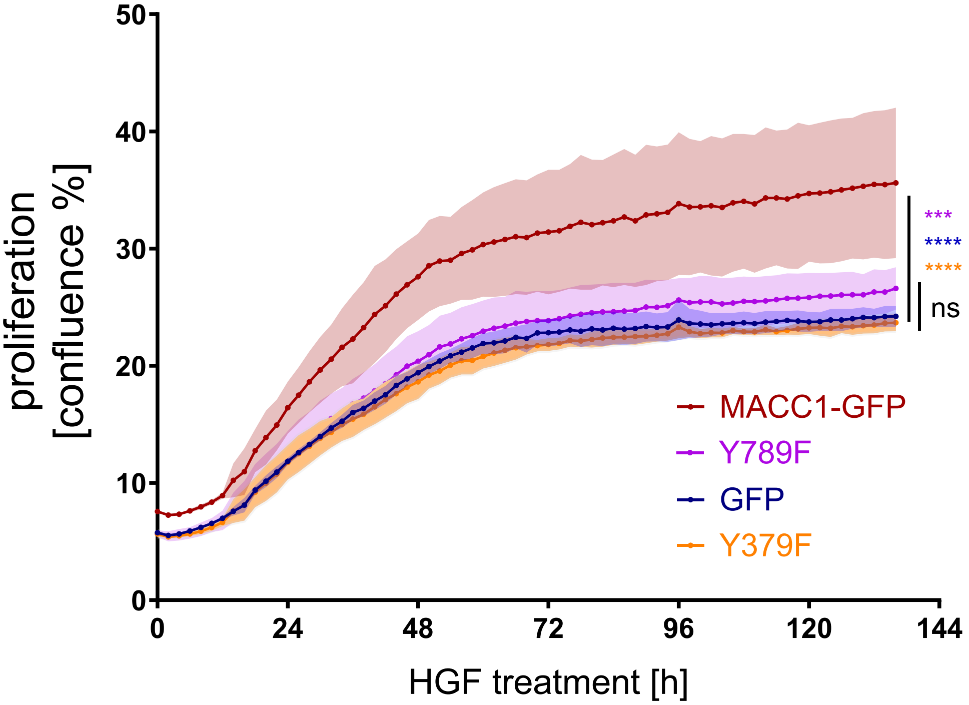

Supplementary Figure S2: *Individual mutation of Y379 and Y789 reduces MACC1-associated proliferation. HCT116 cells expressing GFP, wild-type MACC1-GFP, and the individual MACC1-GFP mutants Y379F or Y789F were analyzed for proliferation following HGF stimulation. Mutation at either site leads to reduced proliferation. Significant results were determined by two-way ANOVA and Tukey's multiple comparison test with a confidence interval of 95% (* = $p < 0.05$, ** = $p < 0.01$, *** = $p < 0.001$). Blue - GFP expressing cells; red - MACC1-GFP expressing cells; purple - MACC1-GFP (Y789F); orange - MACC1-GFP (Y379F) expressing cells.*

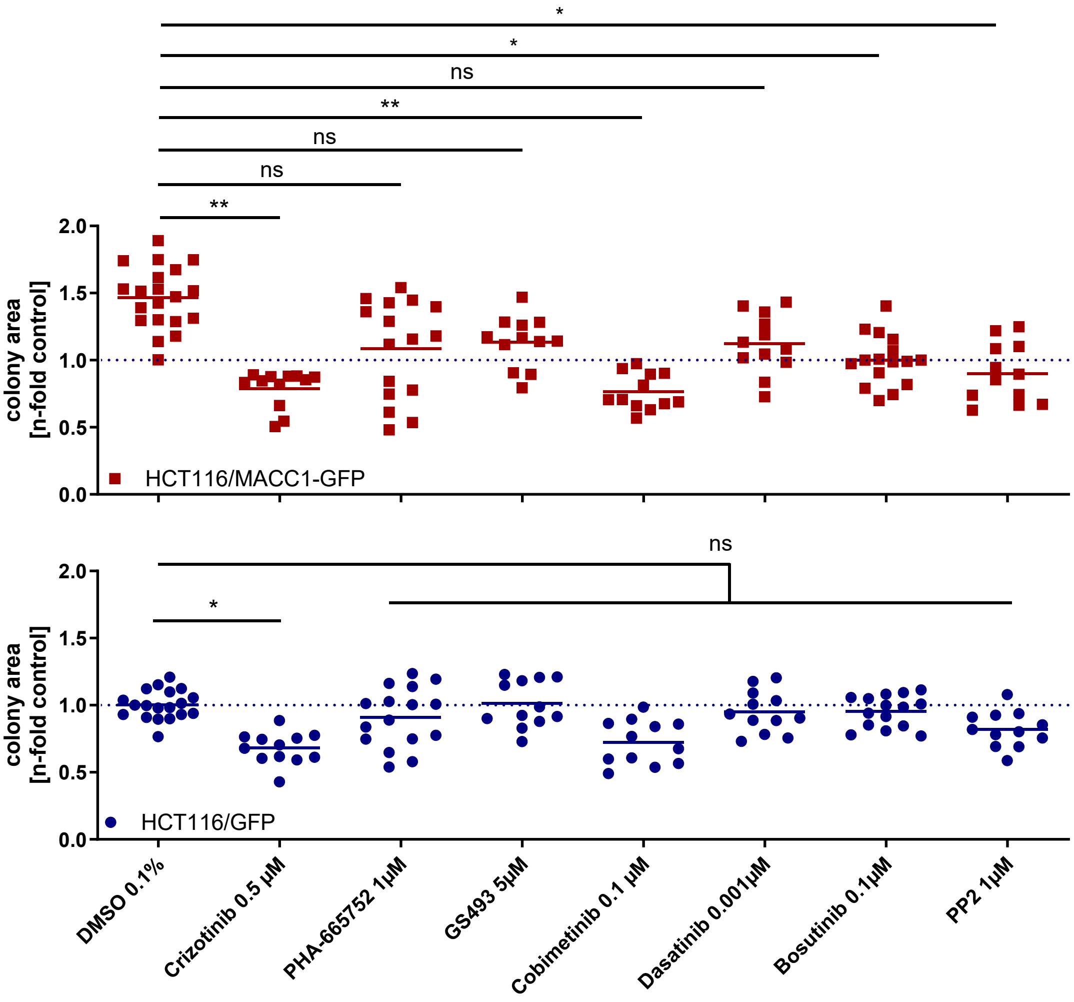

Supplementary Figure S3: *Effect of pharmacological inhibitors on colony formation within GFP control and MACC1-GFP cells. Statistical comparison of colony formation following inhibitor treatment relative to the respective vehicle-treated control was performed separately for HCT116/GFP and HCT116/MACC1-GFP cells. In HCT116/GFP cells, only crizotinib significantly reduced colony formation compared with vehicle treatment, whereas crizotinib, cobimetinib, bosutinib, and PP2 significantly reduced colony formation in HCT116/MACC1-GFP cells. Results represent means of at least three independent experiments. Significant results were determined by one-way ANOVA and Tukey's multiple comparison test with a confidence interval of 95% (* = p < 0.05, ** = p < 0.01, *** = p < 0.001). Blue - GFP expressing cells; red - MACC1-GFP expressing cells.*
